# Whole Liver Derived Acellular Extracellular Matrix for Bioengineering of Liver Constructs: An Updated Review

**DOI:** 10.3390/bioengineering10101126

**Published:** 2023-09-25

**Authors:** Tanveer Ahmed Mir, Alaa Alzhrani, Makoto Nakamura, Shintaroh Iwanaga, Shadil Ibrahim Wani, Abdullah Altuhami, Shadab Kazmi, Kenchi Arai, Talal Shamma, Dalia A. Obeid, Abdullah M. Assiri, Dieter C. Broering

**Affiliations:** 1 Laboratory of Tissue/Organ Bioengineering & BioMEMS, Organ Transplant Centre of Excellence, King Faisal Specialist Hospital and Research Centre, Riyadh 11211, Saudi Arabiatshamma@kfshrc.edu.sa (T.S.);; 2 Department of Medical Laboratory Technology, Faculty of Applied Medical Sciences, King Abdulaziz University, Jeddah 21423, Saudi Arabia; 3 College of Medicine, Alfaisal University, Riyadh 11211, Saudi Arabia; 4 Division of Biomedical System Engineering, Graduate School of Science and Engineering for Education, University of Toyama, 3190 Gofuku, Toyama 930-8555, Japan; maknaka@eng.u-toyama.ac.jp (M.N.); iwa@eng.u-toyama.ac.jp (S.I.);; 5 Department of Child Health, School of Medicine, University of Missouri, Columbia, MO 65212, USA; 6 Department of Clinical Biomaterial Applied Science, Faculty of Medicine, University of Toyama, Toyama 930-0194, Japan

**Keywords:** liver, decellularization, scaffolds, recellularization, tissue and organoids

## Abstract

Biomaterial templates play a critical role in establishing and bioinstructing three-dimensional cellular growth, proliferation and spatial morphogenetic processes that culminate in the development of physiologically relevant in vitro liver models. Various natural and synthetic polymeric biomaterials are currently available to construct biomimetic cell culture environments to investigate hepatic cell–matrix interactions, drug response assessment, toxicity, and disease mechanisms. One specific class of natural biomaterials consists of the decellularized liver extracellular matrix (dECM) derived from xenogeneic or allogeneic sources, which is rich in bioconstituents essential for the ultrastructural stability, function, repair, and regeneration of tissues/organs. Considering the significance of the key design blueprints of organ-specific acellular substrates for physiologically active graft reconstruction, herein we showcased the latest updates in the field of liver decellularization–recellularization technologies. Overall, this review highlights the potential of acellular matrix as a promising biomaterial in light of recent advances in the preparation of liver-specific whole organ scaffolds. The review concludes with a discussion of the challenges and future prospects of liver-specific decellularized materials in the direction of translational research.

## 1. Introduction

Liver diseases are a major concern as they account for millions of deaths annually, and the incidence of hepatic disease is still increasing worldwide [1,2,3]. The liver is the only solid organ in the human body that uses its regenerative capacity to maintain a stable 100% liver-to-body weight ratio at all times, which is necessary to maintain homeostasis throughout the body. Despite its powerful self-regenerative capabilities, liver-linked disorders affect a major population across the globe and represent a significant healthcare and economic burden [2,3,4,5,6]. Liver transplantation is the only live-saving option for patients with end-stage liver disease. Globally, the demand for organs far outstrips the supply. The harsh reality of this disparity in organ need vs adequate availability is that millions of potential patients die on the waiting list [7,8,9]. According to the Organ Procurement and Transplantation Network, in the United States alone, one in four patients seeking a liver transplant either die while waiting (12%), or become too unwell to undergo a liver transplantation operation (13%) [10,11,12,13]. Life-long global pharmacological immunosuppression has greatly reduced episodes of acute graft rejection, leading to considerable success in short-term allograft outcomes. However, their off-target effects can contribute to significant morbidity and mortality [14,15]. Due to the increase in end-stage liver failure and organ supply and demand issues, scientists are exploring alternative treatment options to generate bioengineered 3D tissue grafts and miniaturized versions of organs (organoids) for experimental research and transplant applications.

Generally, traditional laboratory models used in liver research investigations are based on either hepatoma cell lines or primary human hepatocytes grown in two-dimensional monolayer, or whole liver explants. However, these methods lack true in vivo physiological relevance [16,17]. On the other hand, animal models have also been highly instructive in assessing the preclinical safety and effectiveness of new drug candidates. Still, the clinical relevance, ethical issues, and regulatory acceptance of the 3R testing approaches have led the research community toward the development of in vitro methods or alternatives to animal studies [18]. To overcome the weaknesses of 2D monolayer cultures and experimental challenges associated with live animals and humans, the integrated area of cell biology, tissue engineering, and biomaterials science has recently become a research hotspot for the biofabrication of 3D liver tissue/organ-like constructs in vitro [19,20,21,22,23,24,25,26,27]. As outlined by Langer et al. [28], a wide range of tissue engineering methodologies exist, usually combining cell suspensions, supporting scaffolds, and bioactive molecules. Cell sources, extracellular matrix substrate comparable to those of biological tissues (mimicking biological, structural, compositional and organizational properties), and growth-stimulating signals are generally referred to as the tissue engineering triad. Normal cells in biological tissues/organs are anchorage-dependent residing in a 3D microenvironment (absent in 2D cell culture systems). In tissue engineering process, artificially prepared scaffolds and hydrogel microenvironments serve as temporary structural frameworks and provide unique opportunities for applied cells to attach, proliferate, differentiate, and migrate in biomimetic 3D microenvironments during various growth, development, and maturation stages [26,28,29,30,31].

Over the years, researchers have developed biocompatible materials to create scaffolds and hydrogels comparable to the native microenvironment of target tissues and organs, either through materials chemistry approaches or extraction/modification of naturally occurring materials [32,33]. Biomaterials (e.g., synthetic, natural or bioconjugations of both) applied in developing tissue-engineered liver constructs are primarily categorized according to their geometrical configuration, chemical composition, physical or mechanical integrity, biofunctionality and biodegradability. Structural integrity and biophysicochemical cues of the natural or synthetic polymeric scaffold matrices play important roles in controlling cellular dynamics, polarity, cell–cell communication, and crosstalk events [34,35,36,37,38]. More importantly, artificial scaffolding materials biodegrade while serving as biomimicking 3D microenvironments for cellular growth, neo-tissue formation, and maturation. Using the structural and functional insights gained from natural extracellular matrices as a blueprint, a myriad of biomaterials has been developed. However, to date, there is no natural or synthetic material that can fully reproduce all of the multidomain macromolecular dynamic properties of the liver-specific extracellular matrix in vitro [39,40,41]. Decellularization is therefore a highly sought after technique for obtaining native acellular microenvironments from xenogeneic or allogeneic tissue sources and is currently being used in both basic and translational research to generate physiologically relevant 3D in vitro tissue constructs [42,43,44,45,46,47,48,49]. The unique architectural, topological and functional cues of organ-specific scaffolds make them interesting for inducing desirable cell-specific responses and downstream applications such as high throughput screening, disease modeling and hepatoxicity testing. This review sheds light on the versatility of decellularized materials derived from the mammalian liver to develop whole liver scaffolds. Finally, we summarized the current limitations of decellularized materials and future directions.

## 2. Hepatic Extracellular Microenvironment and Its Key Functions

The liver extracellular matrix is a highly intricate three-dimensional meshwork of fibers in which hepatic cells reside in an orderly fashion [50]. Essential components of the liver ECM include a variety of macromolecules, including biopolymers, glycosaminoglycans, proteoglycans, glycoproteins, numerous growth factors, cytokines and other matrix-bound bioactive nanovesicles. Collagen types I, III, IV, V, VI, VII, and VIII, fibronectin, laminin and elastin are broadly classified as the most essential structural and adhesive components of the ECM [51]. However, since these biomolecules are not evenly distributed throughout the organ, the structure of the ECM varies greatly from one specific region of the tissue to another in terms of the proportion and arrangement of its components. In a healthy normal liver, fibrillar types of collagen (Collagen I, III and V) are abundantly localized in hepatic capsules, around portal stroma areas, the perisinusoidal space and fibroid tissue [52,53]. Type IV collagen and laminin work in a tandem network to make up the basement membrane of blood vessels and bile ducts. Type IV collagen, along with other non-fibrillar proteins such as laminin, form a low-density basement membrane-like matrix along the hepatic sinusoids, around the vessels of the portal tract and bile ducts [54,55,56,57,58,59].

**Figure 1:**
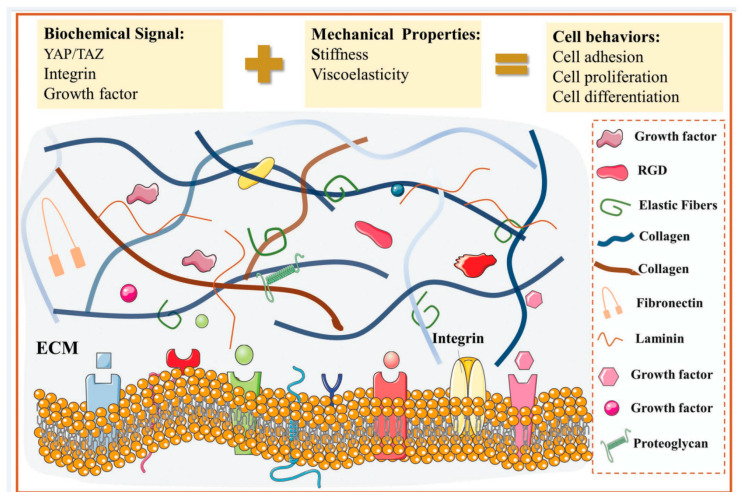
Schematic diagram of extracellular matrix components showing biochemical signaling molecules. Figure 1 is reproduced with copyright permission from [66], Wiley.

The non-cellular components of the liver extracellular matrix have traditionally been appreciated only as a dynamic and inert structural network that provides a supportive scaffold for stable distribution and orientation of cells within the tissue/organ. However, studies over the past decade have revealed that the physiological relevance of the liver extracellular matrix extends beyond simple skeletal protection of cells and surrounding tissues to several other fundamental biological and functional cues that regulate various intra- and intercellular activities. Any unfavorable qualitative and quantitative disruption in the extracellular matrix by various pathogenicity factors can directly affect the structural foundation, histology, anatomy, and physiology of the liver, paving the way for the initiation and progression of liver diseases. Nevertheless, preserving the native hepatic ECM structure is critical for inducing or controlling many cellular processes essential for tissue regeneration through inherent physical, chemical, and biological cues. Because ECM operates as a communication liaison between cells in the tissue/organ, it is significantly important to accurately reconstitute the multifactorial biophysicochemical properties of the native liver’s extracellular matrix when creating implantable tissue engineered constructs [60,61,62,63,64,65]. Schematic representations of the ECM composition and its biochemical or mechanical properties are shown in Figure 1.

## 3. Development of Liver Specific Decellularized Biomaterials for Liver Tissue Engineering

**Figure 2:**
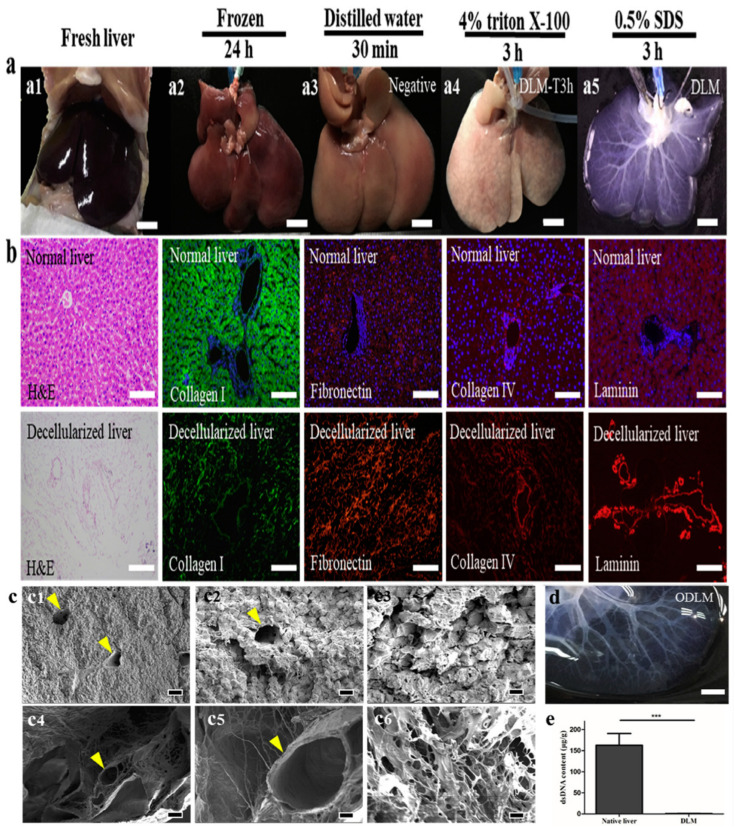
Macroscopic images for the preparation and characterization of native and decellularized rat liver (**a**). (**a1**) Fesh liver, (**a2**) frozen liver-after 24 h, (**a3**) liver perfused with distilled water over 30 min through both portal vein and bile duct systems, (**a4**) liver perfused with 4% TritonX-100 solution over 3 h and (**a5**) liver perfused with 0.5% SDS solution over 3 h. (**b**) Cross sections of histological and immunofluorescence images of native and decellularized livers stained with hematoxylin-eosin, fibronectin (red), collagen I (green), laminin (red), and collagen IV (red) showing the overall structure, sulfated GAG, and collagen, respectively. (**c**) Scanning electron microscopy images showing ultrastructure of normal and decellularized livers treated with 0.4% Triton and 0.5% SDS based protocols. SEM images of (**c1**–**c3**) normal liver and (**c4**–**c6**) decellularized liver. Intact and smooth vessel wall (**c5**) and extracellular matrix parenchyma (**c6**) with hepatocyte-sized free space in the decellularized liver matrix can be clearly observed. Yellow triangles in indicate the vessel walls native and decellularized liver. (**d**) Appearance of over perfused decellularized liver left lobes, with vasculature preserved. (**e**) Confirmation of DNA removal native and decellularized rat liver. Data are expressed as means ± SD (n = 3). *** *p* < 0.001. Figure 2 is adopted with copyright permission from [136], Elsevier.

The significance of the extracellular matrix and its biophysicochemical role in so many fundamental biological processes has stimulated substantial interest in the formulation of next generation biomaterials. Native liver-derived acellular materials are considered the most biomimetic, reliable and instructive substrates for unlocking the inherent regenerative potential of locally damaged liver tissue compared to other natural and synthetic materials [67,68,69,70,71,72]. Key features of native extracellular matrix, such as high biocompatibility, low immunogenicity, and excellent biodegradability are extremely difficult to mimic with synthetic materials [73,74,75,76,77,78,79,80,81,82,83,84,85,86,87,88,89]. Acellular materials of biological origin are usually obtained through decellularization, which ideally refers to the complete removal of cellular components and genetic materials (DNA and RNA) using various agents. Various decellularization methodologies (physical, chemical, enzymatic) have been developed to isolate all cellular and immunogenic components from whole liver/tissue slices in order to obtain liver tissue-specific bioactive materials that replicate the maximum dynamic scaffolding integrity and biomolecular compositions provided by the native extracellular matrix proteins in vivo [90,91,92,93,94,95,96,97,98,99,100,101,102,103,104,105,106,107,108,109,110,111,112,113,114,115]. The importance of the bioactivity of dECMs in liver tissue/organ bioengineering has been highlighted in many studies [116,117]. Although experimental strategies for decellularization of liver tissue/entire liver have improved significantly and several relevant studies have been published, the need for a gold standard tissue-independent decellularization protocol still prevails. This is because each individual donor source (animal or human) possesses distinct features in terms of size of the tissue/organ, eidonomy, anatomical architecture, cellular composition, extracellular matrix organizations, stiffness and quantity of the interlocking ingredients. Accumulated knowledge has revealed over the past decade that the relevant physicochemical characteristics and mechanobiological profiles in the preserved components of decellularized liver biomaterials vary and generally depend on the method of decellularization (Figure 2). For these reasons, when performing decellularization treatments, it is essential to recognize that one experimental protocol may not yield efficient outcomes and the development of the more advanced decellularization–recellularization technologies offer the opportunity to obtain reliable preclinical results or development of clinical grade tissue engineered products [48,118,119,120,121,122,123,124,125,126,127,128,129,130,131,132,133,134,135,136].

## 4. Liver-Derived Acellular Matrix as a Platform for Whole Organ Bioengineering

Over the past decade, several research groups have been able to fully decellularize simple tissues to whole-livers and demonstrate the ability to repopulate cells into acellular templates for experimental biology research and preclinical applications [137,138,139,140,141,142]. One of the first studies on bioengineering the whole liver using decellularization and recellularization approaches was reported by Uygun et al. [142]. The authors decellularized ischemic rat livers using sodium dodecyl sulfate (SDS) and performed washout perfusion through the portal vein for 72 h. The characterization results revealed that the decellularized scaffold retained the ultrastructural components of the liver extracellular matrix (collagen types I and IV, fibronectin, and laminin β1) and microvascular network. To validate the effectiveness of the acellular liver scaffold, the authors performed recellularization with rat primary hepatocytes (four injections of 5 × 10^6^) through the portal vein. The entire liver scaffold supported efficient cell engraftment with 96.5% ± 3.6% efficiency. Initially, the injected cells were adhered around large veins, but over the next few days, the cells were found to be distributed throughout the matrix. During the experiment period, approximately 20% of cells were damaged due to microenvironmental perturbations and apoptosis. Interestingly, functional analysis revealed elevated levels of albumin, urea, UDP-glucuronyltransferase 1 family, polypeptide A1, and glucose 6-phosphatase. Expression levels of cytochrome P450 enzymes were reported to be similar to those found in normal livers. The researchers also experimented with the addition of microvascular endothelial cells to the recellularized structures and succeeded in aligning the vasculature in three days. Preserving vascular network allowed the transplantation (heterotopic) of the recellularized liver as auxiliary liver in rats through the arterialization of the portal vein.

Around the same year, Shupe et al. [143] also reported a perfusion strategy using Triton X-100 in combination with 0.1% SDS to achieve more efficient decellularization results in a less time-consuming manner. The authors performed perfusion mediated recellularization using rat liver progenitor cells (WB344) through the inferior vena cava (IVC) route. Although, long-term analyses were not performed, the researchers observed the migration of the cells from the vessels to the center of the acellular matrix. This was followed by the active exploration of decellularization–recellularization technology for liver tissue engineering research. For example, Gessner et al. [144] developed a liver scaffold from rats by perfusing the detergent fluids through the portal vein route. The authors perfused the liver for 30 min–1 h using a dewaxing buffer (36 U/L phospholipase A2 in 1% sodium deoxycholate) in order to remove the biological membranes (plasma and nuclear membrane). To maintain the biophysicochemical characteristics of the native organ, the liver was also infused with a high-salt buffer that helped keep collagen insoluble and preserved the cytokines and growth factors. Nucleases (DNases and RNases) were exposed to the decellularized scaffold to remove any remaining nuclear material in the acellular structural framework. Surface topography captured using scanning electron microscopy (SEM), confirmed that extracellular components were preserved at a comparable level to normal liver tissue. Maintenance of the microvasculature integrity allowed the reseeding of human hepatoblast-like cells (Hep3B cells). The recellularized structure with a cell seeding density of 1.3 × 10^8^ cells was maintained in bioreactors for up to 14 days. At the termination of the experiment, lobes of the recellularized matrix framework were used for assessing the cell distribution using immunofluorescence, immunohistochemistry and scanning electron microscopy imaging. The results showed that engrafted cells exhibited proliferation potential (Ki67 staining) without showing the signs of apoptosis. Additionally, biomarkers such as albumin and EpCAM were expressed, but the expression levels were directly dependent on the localization of the attached cells. In the same year, a Japanese research group led by Yagi et al. [145] reported an improvement in the decellularization protocol for porcine liver. The authors performed decellularization in larger animal experiments with the intent of generating large sized organs similar to the human liver, an important step toward engineering whole organs for clinical applications. The results showed that the morphological and structural components of the liver were well preserved after undergoing decellularization procedures. The presence of growth factors (hepatocyte growth factor, basic fibroblast growth factor, insulin-like growth factor 1, and vascular endothelial growth factor) crucial for maintaining a healthy niche of hepatic cells was also analyzed. Still, the growth factors in the acellular matrix were significantly lower than in normal liver tissue. Prior to the recellularization, the authors sterilized the scaffold with ultraviolet irradiation and performed a multi-step infusion of hepatocytes (1 × 10^9^) through the portal vein. Albumin staining results (after 4 and 7 days of culture) revealed that the hepatocytes remained mostly in the portal vein for the first 24 h, then engrafted and migrated to the surrounding liver parenchymal region. Interestingly, after 4 days of the perfusion culture incubation, grafted hepatocytes showed albumin expression levels comparable to those of normal livers; however, long-term functionality analysis showed that expression levels decreased significantly only after day 7.

Around 2015, researchers started to explore new strategies for reestablishing the vascular network within the decellularized liver scaffold. To investigate the vasculature reconstruction concept, Ko et al. [146] utilized Triton X-100 and 0.1% ammonium hydroxide as detergents for the perfusion decellularization of porcine liver through the portal vein and hepatic artery routes. To understand the reestablishment of the vascular network, the authors bioconjugated the acellular scaffold with anti-endothelial cell specific antibodies by employing 1-ethyl-3-[3-dimethylaminopropyl] carbodiimide hydrochloride/and N-hydroxysuccinimide ester chemistry. This was the first report demonstrating a strategy to maximize the coverage of acellular vessel walls with GFP protein (MS1)-expressing vascular endothelial cells. As a result, the endothelium adhered uniformly throughout the vasculature, reached the capillary bed of the scaffold and greatly reduced platelet adhesion during blood perfusion in vitro. The authors further validated the vascular functionality by transplanting the reendothelialized livers using a pig model. On day 1 after heterotrophic transplantation, the vascular patency of the scaffolds was examined by ultrasound imaging and radiographic fluoroscopy techniques. The recovered scaffolds were examined by histological (H&E) and immunohistochemical (platelet immunostaining) analysis. The results demonstrated that the reendothelialized scaffolds were able to withstand physiological blood pressure and maintained blood flow within the bioengineered constructs for 24 h.

To further improve vascular reconstruction and enhance hepatic functions in bioengineered livers, Hussain et al. [147] hypothesized that a mixed heparin–gelatin coating on the scaffold would facilitate optimal antithrombotic management and enhance endothelial cells attachment as well as migration on vascular spaces within decellularized livers by exploiting gelatin’s multiple integrin binding sites. To evaluate the effect of reendothelialization on parenchymal cells, the authors co-cultured hepatocellular carcinoma (HepG2) cells and ECs. Finally, recellularized scaffolds were heterotopically transplanted in a porcine model. The overall results showed that the heparin–gelatin coating improved ex vivo blood perfusion when compared to non-coated frameworks. This was followed by a study published by Devalliere et al. [148], where the authors employed a different strategy for enhancing reendothelialization and manipulating endothelial cell attachment in decellularized rat liver scaffolds. In order to facilitate endothelial cell binding to vessel walls, the authors genetically fused elastin-like peptide (ELP) to five internal peptide sequences (REDV) of the CS5 segment of fibronectin. The linkages of the cell-binding domain REDV via REDV–ELP coupling enhanced the attachment, proliferation and spreading of endothelial cells within the acellular structure. The results showed that modification of the scaffold with REDV–ELP resulted in the formation of a uniform endothelial lining of the vasculature and a clear decline in platelet adhesion to the substrate.

**Figure 3:**
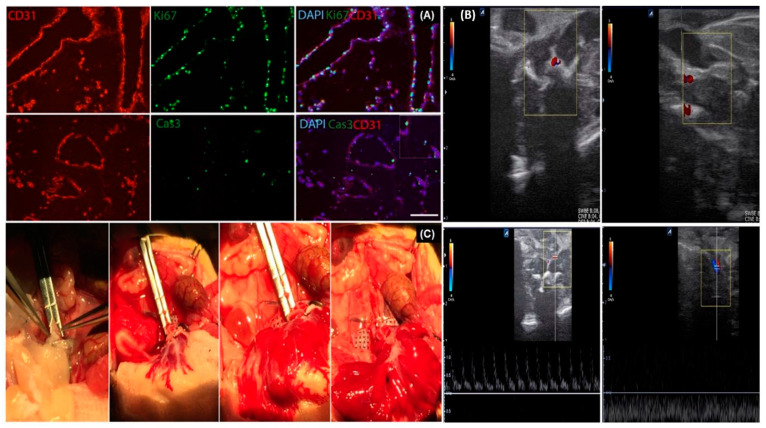
Decellularized rat liver scaffold after reendothelialization. (**A**) Adhesion of endothelial cells to the vascular walls and infiltration towards the extravascular. (**B**) Anastomosis of the PV and IVC of the reendothelialized liver scaffold to the abdominal aorta and IVC of the recipient rat. (**C**) Ultrasound imaging showed the blood flow into the transplanted scaffold 8 days post-transplantation. Figure 3 is reproduced with copyright permission from [149], Wiley.

Joining forces in the development of potential preclinical models, our research laboratory, Meng et al. [149], performed whole liver decellularization and employed a different approach for the reconstruction of vasculature in rat liver derived scaffolds. The authors hypothesized that the infusion of gelatin-encapsulated cells would improve reendothelialization. Results showed that perfusion of immortalized endothelial cells-encapsulated in gelatin-based cocktails facilitated the retention of large numbers of cells in the recellularized scaffolds. Recellularized liver scaffolds were transplanted heterotopically in a rat model. Observations using doppler ultrasound waves showed that blood was actively flowing within the reendothelialized liver scaffold on day 8 after transplantation (Figure 3). In addition, platelet aggregation and thrombus formation were observed in the vascular lumen of the reendothelialized liver scaffold on day 8 post-transplantation.

**Figure 4:**
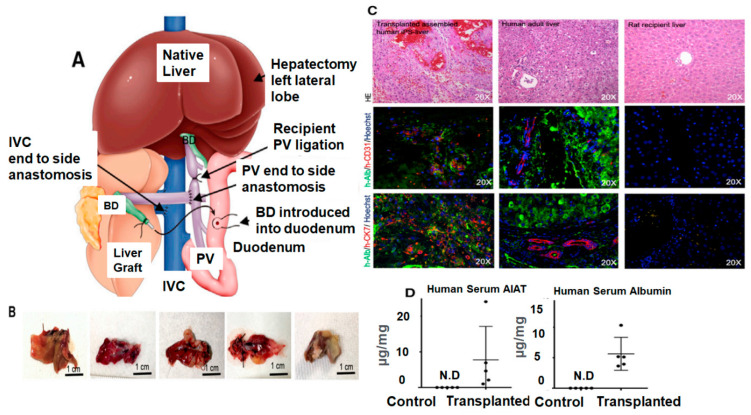
Transplantation (auxiliary) of the bioengineered human liver graft recellularized with induced pluripotent stem cells (iPSCs). (**A**) Schematic description of the surgical techniques for the transplantation of auxiliary liver grafts or human bioengineered liver grafts: (1) after right nephrectomy, (2) PV and IVC were exposed. (3) IVC anastomosis (end to side). (4) PV anastomosis (end to side). (5) After reperfusion. (6) Before closing abdomen. (**B**) Microscopic images of the engineered liver graft three–four days post-implantation. (**C**) immunofluorescence staining of recellularized auxiliary graft post-transplantation (left), compared to human adult liver tissue (middle), and rat recipient liver (right). H&E, hematoxylin and eosin; h-ALB, human-specific albumin; h-CD31, human-specific CD31; h-CK7, human-specific cytokeratin 7. Sections were counterstained with Hoechst (blue stain). (**D**) The serum concentration of human specific A1AT and human-specific ALB was measured by ELISA at day 4 post-transplantation. Abbreviation: N.D: Not determined. Figure 4 is adopted with copyright permission from [150], Elsevier.

Recently, Takeishi et al. [150] took advantage of the characteristics of human induced pluripotent stem cells (iPSCs) to create functional bioengineered liver. In the first attempt, the researchers improved the liver decellularization process by using a 30-fold lower concentration of Triton X-100 than previously published protocols, and then assessed the biological and biomechanical features of the dECM components using a differential scanning calorimetry technique. Subsequently, they optimized the protocols for generating the human iPSC-derived hepatocytes, iPSC-derived cholangiocytes, and iPSC-derived endothelial cells for recellularization of the decellularized livers with parenchyma, biliary system, and vascular spaces. Interestingly, when researchers transplanted bioengineered livers into an immunocompromised rat model, they were able to maintain function for four days (Figure 4). Many researchers have published similar studies, but what makes this multistep protocol unique from others is their ability to repopulate not only the parenchymal cells, but also the vasculature and biliary network. The authors illustrated that an organ-specific acellular scaffold showed marked improvement in the differentiation of specialized liver cell lineages (hepatocytes, cholangiocytes, and vascular endothelial cells) in the pertinent parenchymal and non-parenchymal structures. Although, iPSC-derived hepatic cells alleviate the procurement limitations of primary human cells, they not only mimic the fetal rather than the adult cells’ phenotype, but are also functionally immature. Due to low differentiation functions, engineered liver acellular structures recellularized with iPS cell-derived lines exhibited immaturity, but urea production analysis markedly improved [151,152,153]. Overall, functional analysis revealed that the decellularized liver scaffolds repopulated with different cell types derived from human iPSCs, showed adequate liver function both in vitro and in vivo. Although the recellularized liver remained functional in vivo for four days after auxiliary transplantation into immunocompromised rats (interleukin 2rg^−/−^), the overall strategy was not 100% effective in repopulating the bile ducts or vascular tree. Additionally, the rats had to undergo a right native nephrectomy to create space for the allograft as well as a left lateral lobectomy of the native liver to induce regeneration. The final outcomes were unsatisfactory because by four days, two rats had developed infection with poor blood flow throughout the graft, one developed portal vein thrombosis, and the other two developed intestinal ischemia.

In recent years, similar decellularization and recellularization techniques have been investigated for continuous perfusion of bioengineered livers in a large animal model, but these efforts have focused primarily on endothelial cell-based revascularization. For example, Shaheen et al. [154] reported an improved technique with the capability to functionally reendothelialize the vasculature of a human-sized acellular liver scaffold using human umbilical vein endothelial cells (HUVECs) in a large animal recovery model while maintaining continuous perfusion. A follow-up study carried out by the same group, Anderson et al. [155], demonstrated the seeding and engrafting of primary porcine hepatocytes into bioengineered liver (BEL) scaffolds that had been previously reendothelialized with HUVECs. The results showed that bioengineered livers were functionally competent enough for the production of albumin, synthesis of urea and ammonia detoxification that indicated the presence of a functional hepatocyte compartment. Furthermore, bioengineered livers delayed ammonia accumulation during in vivo perfusion in a porcine model of surgically induced acute liver failure. After graft removal, bioengineered liver parenchyma was found to maintain canonical endothelial and hepatocyte biomarkers. As Shaheen et al. [154] demonstrated in their heterotopic transplantation of endothelialized liver constructs into an immunosuppressed large animal model, the pig survived for approximately 15 days post-transplantation, but the reported technique still possesses several technical and practical limitations that must be resolved. The main problem with this study, however, was that only 11.9% of the allograft portal veins were found to be patent at the study end point. Other important issues that need to be resolved include long-term maintenance of transplant functions in large animals, recellularization of multiple cell types, human liver-sized cell populations, and application to preclinical large animal models with liver-related health disorders. 

**Figure 5:**
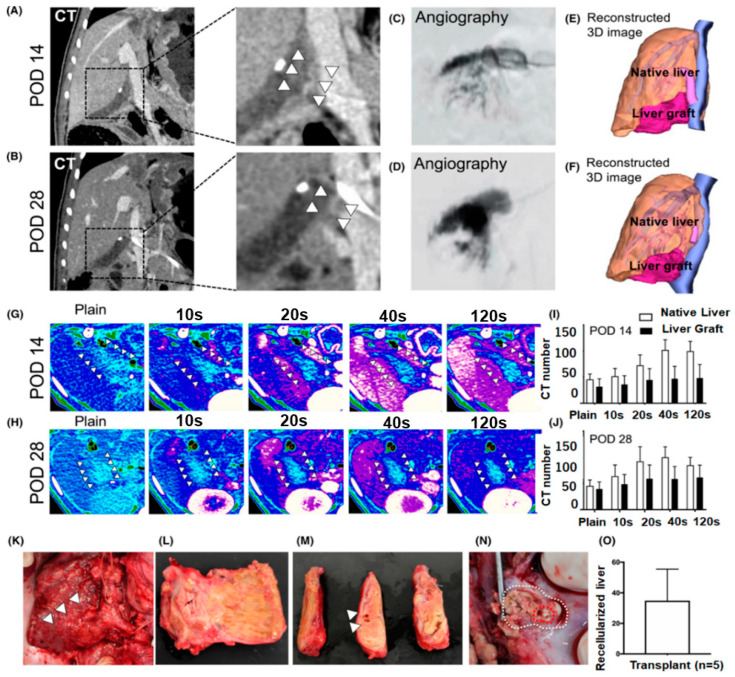
Engraftment (in vivo) of the bioengineered liver graft at different time points (postoperative days 14 and 28). (**A**,**B**) CT images of the transplanted graft on postoperative 14 (**A**) and postoperative 28 (**B**). Angiography through the intraportal infusion catheter on postoperative 14 (**C**) and postoperative 28 (**D**). Reconstructed three-dimensional CT image of the remnant native liver and the transplanted graft on postoperative 14 (**E**) and postoperative 28 (**F**). Time courses of the CT images for the transplanted graft on postoperative 14 (**G**) and postoperative 28 (**H**), Blue tube depicts IVC and HV, and pink tube depicts PV. Time course of the mean CT numbers for the graft and remnant native liver on postoperative 14 (**I**) and postoperative 28 (**J**). Intraoperative images of the graft on postoperative 28 (**K**,**L**). Macroscopic images of the procured graft on postoperative 28 (**M**). The arrowheads indicate PV in the graft. Macroscopic image of the stenosed anastomosis site of the procured graft on POD 28 (**N**). Patency rate was estimated to be approximately 11.9%. The dashed red and white lines show the anastomosis site and patent area, respectively. Weight of the procured graft on postoperative 28 (**O**). Figure 5 is adopted with copyright permission from [156], Elsevier.

The most significant advancement to date, at least in terms of successful hepatic vascular perfusion of repopulated scaffolds or in vivo perfusion duration, has been made by Higashi et al. [156] who seeded decellularized whole pig livers with HUVECs. The authors found that they were able to perfuse the heterotopically implanted scaffolds successfully for up to 20 days. Their study was primarily focused on (i) establishing human-sized bioengineered livers, (ii) optimizing protocols by recellularization of multiple cell types, (iii) optimizing protocols for efficient transplantation of bioengineered liver grafts in large animal models with liver dysfunction, and more importantly (iv) to improve the post-transplant survival of the bioengineered graft in pigs with induced liver failure. Interestingly, the authors found that the auxiliary bioengineered liver graft improved liver function and increased the expression of liver-specific genes over 28 days (Figure 5). This was the first study of its kind to present 28 days of post-transplant evaluation of a bioengineered liver graft using a preclinical large animal model.

## 5. Current Challenges and Future Directions

Over the past several years, decellularized materials derived from liver from multiple mammalian sources (e.g., rat, mouse, pig, human) have been used for tissue and organ bioengineering applications. It is well documented that the decellularized liver extracellular matrix offers a unique amalgamation of ultrastructural connectivity and inherent biochemical features that allow dECM to be exploited for cell culture, adhesion, proliferation, differentiation, migration, and maintenance of morphologic integrity of different hepatic cell types during subsequent recellularization procedures. Broadly speaking, several aspects of decellularization and recellularization technology are immediately quite appealing, and successful translation and clinical-grade bioengineered organ supply to patients with end-stage liver disease could ultimately transform the scenario of the transplant program, worldwide. The main challenge in decellularization/recellularization technology is the inability to efficiently generate fully hemocompatible and endothelialized whole organ (liver)-like constructs containing multiple cell lines with an intact and healthy vasculature comparable to that of the native organ. It is evident that converging various advanced interdisciplinary research approaches may one day generate volumetric liver tissue or autologous bioengineered organ equivalents for deployment in clinical settings for transplantation purposes [157,158,159].

Although preliminary findings are quite encouraging, this research domain is still in its infancy. Notably, decellularized matrix obtained from native liver displays several key changes, including the unintended removal of small molecules and incomplete recapitulation of the native organ. To motivate further developments, there are several other critical issues that need to be resolved immediately, such as the effect of different decellularization methods on the variation of obtained liver acellular matrix, preservation or reconstruction of native vasculature networks, homogeneous repopulation of the entire scaffold and its hidden compartments with autologous cells, and sustained diffusion of the nutrients and oxygen both in vitro and in vivo [142,150,154]. Other important issues that need to be addressed include employment of multiple cell types, development of a universal seeding protocol, ex-vivo maintenance of repopulated livers, real-time monitoring and preventing total arterial and venous thrombotic events, and the reproduction of histotypic liver microstructure and zonation.

As explained above, several research laboratories have explored various decellularization and recellularization approaches for whole liver bioengineering. Indeed, published studies underpin forward-looking options of the combined implementation of various convergent approaches based on cell biology/pluripotent stem cell biology, surface modification chemistry, and bioengineering principles. The recellularized whole liver grafts generated by the coupling of recellularization and stem cell-based technologies have generally been transplanted as auxiliary organs. All animal models in which auxiliary transplantation of liver grafts was performed, exhibited several limitations. The main obstacle was the lack of advanced technical and scientific methodologies to address issues related to hepatic arterial blood supply. In general, the auxiliary grafts are supplied with blood from the portal vein, which may not provide adequate oxygenation and potentially limit the functionality. Similarly, the absence of a bile duct system inhibits external bile drainage from the graft. In such a scenario, bioengineered liver allografts cannot effectively handle bile production and secretion, which may inevitably lead to complications. In addition, the need for intra-abdominal space for optimal graft placement, the need for various cell types to repopulate the liver graft, cellular immunogenicity, and the risk of developing thrombosis, are also some of the burning issues. To improve graft functionality in vivo, all of the above-mentioned issues related to auxiliary liver transplantation in animal models need to be addressed urgently [145,150,154]. Therefore, additional work based on multidisciplinary approaches is needed to realize the potential of this technology for the bioengineering of fully functional and clinically transplantable humanized liver bioequivalents. Nevertheless, the published reports are inspirational steps toward achieving this unforeseen goal.

## 6. Conclusions

Despite the advancements in decellularization and recellularization methodologies, establishment of completely functional liver grafts/in vitro models remain a huge challenge. Our overview of the various decellularization approaches for obtaining liver specific homogenous acellular scaffolding systems highlights the fundamental obstacles associated with recapitulating the liver’s extracellular microenvironment in vitro. We also demonstrated that the cell culture platforms based on organ-specific decellularized biomaterials exhibit several key obstacles that need to be resolved immediately. For example, there is still no consensus about which protocol is universal for obtaining high grade decellularized matrix without losing its essential properties or the best recellularization culture procedures. Therefore, significant advancements are necessary especially for developing novel detergent-free protocols, establishing aseptic decellularization workstations, and bioprocess engineering tools for controlling batch-to-batch variability related issues. As a whole, to improve functionality, immunogenicity, maturation and sustainably of the decellularized grafts for both in vitro and in vivo transplantation applications, researchers must address the critical obstacles associated with production of organ-specific materials with desired structural, functional and mechanical properties. Other important issues that need to be intensively addressed to realize recellularization technology include a proper selection of cell sources or appropriate cell types (parenchymal and nonparenchymal cells), quantity control of the cultures, reseeding endothelial cells to cover the endothelial lining and vascular spaces, optimization of recellularization routes, and ensuring efficient blood flow. Further efforts in technological and materiobiological innovation will allow researchers to explore alternative approaches to providing tissue/organ substitutes from bioengineered sources that will have profound implications for studying liver-related pathologies or regeneration of a fully functional organ replacement. However, there are additional roadblocks that must be addressed in the future, including the availability of the organs, the need for instrumentation to maintain and perform the entire decellularization and recellularization procedure, overall cost, clinical trials, and regulatory approvals.

## Data Availability

Not applicable.

## References

[1] Asrani S.K., Devarbhavi H., Eaton J., Kamath P.S. (2019). Burden of liver diseases in the world. J. Hepatol..

[2] Lotto J., Stephan T.L., Hoodless P.A. (2023). Fetal liver development and implications for liver disease pathogenesis. Nat. Rev. Gastroenterol. Hepatol..

[3] Solhi R., Lotfinia M., Gramignoli R., Najimi M., Vosough M. (2021). Metabolic hallmarks of liver regeneration. Trends Endocrinol. Metab..

[4] Taub R. (2004). Liver regeneration: From myth to mechanism. Nat. Rev. Mol. Cell Biol..

[5] Gilgenkrantz H., Collin de l’Hortet A. (2018). Understanding Liver Regeneration: From Mechanisms to Regenerative Medicine. Am. J. Pathol..

[6] Campana L., Esser H., Huch M., Forbes S. (2021). Liver regeneration and inflammation: From fundamental science to clinical applications. Nat. Rev. Mol. Cell Biol..

[7] Dienstag J.L., Cosimi A.B. (2012). Liver transplantation—A vision realized. N. Engl. J. Med..

[8] Bodzin A.S., Baker T.B. (2018). Liver Transplantation Today: Where We Are Now and Where We Are Going. Liver Transplant..

[9] Wilke T.J., Fremming B.A., Brown B.A., Markin N.W., Kassel C.A. (2022). 2020 Clinical Update in Liver Transplantation. J. Cardiothorac. Vasc. Anesth..

[10] Organ Procurement and Transplantation Network. https://optn.transplant.hrsa.gov/data/view-data-reports/national-data/#.

[11] Norah A.T., Claire F., Marina B., Michael C., Julie H. (2023). Liver Transplantation 2023: Status Report, Current and Future Challenges. Clin. Gastroenterol. Hepatol..

[12] Samuel D., Coilly A. (2018). Management of patients with liver diseases on the waiting list for transplantation: A major impact to the success of liver transplantation. BMC Med..

[13] Daniel Z.V., Pilar L.L., Peter K., Giuliano T. (2017). Fighting Mortality in the Waiting List: Liver Transplantation in North America, Europe, and Asia. Ann. Hepatol..

[14] Dery K.J., Górski A., Międzybrodzki R., Farmer D.G., Kupiec-Weglinski J.W. (2021). Therapeutic Perspectives and Mechanistic Insights of Phage Therapy in Allotransplantation. Transplantation.

[15] Wood K.J., Shankar S., Hester J., Issa F. (2019). Concepts and Challenges in Organ Transplantation: Rejection, Immunosuppression, and Tolerance. Clinical Immunology.

[16] Du Y., Han R., Ng S., Ni J., Sun W., Wohland T., Ong S.H., Kuleshova L., Yu H. (2007). Identification and characterization of a novel prespheroid 3-dimensional hepatocyte monolayer on galactosylated substratum. Tissue Eng..

[17] Ng S., Han R., Chang S., Ni J., Hunziker W., Goryachev A.B., Ong S.H., Yu H. (2006). Improved hepatocyte excretory function by immediate presentation of polarity cues. Tissue Eng..

[18] Smith A.J., Lilley E. (2019). The Role of the Three Rs in Improving the Planning and Reproducibility of Animal Experiments. Animals.

[19] Lee J.S., Cho S.W. (2012). Liver tissue engineering: Recent advances in the development of a bio-artificial liver. Biotechnol. Bioprocess Eng..

[20] Zhang J., Zhao X., Liang L., Li J., Demirci U., Wang S. (2018). A decade of progress in liver regenerative medicine. Biomaterials.

[21] Müller F.A., Sturla S.J. (2019). Human in vitro models of nonalcoholic fatty liver disease. Curr. Opin. Toxicol..

[22] Mir T.A., Iwanaga S., Kurooka T., Toda H., Sakai S., Nakamura M. (2019). Biofabrication offers future hope for tackling various obstacles and challenges in tissue engineering and regenerative medicine: A Perspective. Int. J. Bioprint..

[23] Bomo J., Ezan F., Tiaho F., Bellamri M., Langouët S., Theret N., Baffet G. (2016). Increasing 3D Matrix Rigidity Strengthens Proliferation and Spheroid Development of Human Liver Cells in a Constant Growth Factor Environment. J. Cell. Biochem..

[24] Lauschke V.M., Hendriks D.F.G., Bell C.C., Andersson T.B., Ingelman S.M. (2016). Novel 3D Culture Systems for Studies of Human Liver Function and Assessments of the Hepatotoxicity of Drugs and Drug Candidates. Chem. Res. Toxicol..

[25] Arai K., Yoshida T., Okabe M., Goto M., Mir T.A., Soko C., Tsukamoto Y., Akaike T., Nikaido T., Zhou K. (2017). Fabrication of 3D-culture platform with sandwich architecture for preserving liver-specific functions of hepatocytes using 3D bioprinter. J. Biomed. Mater. Res. A.

[26] Bhatia S.N., Underhill G.H., Zaret K.S., Fox I.J. (2014). Cell and Tissue Engineering for Liver Disease. Sci. Transl. Med..

[27] Dash A., Inman W., Hoffmaster K., Sevidal S., Kelly J., Obach R.S., Griffith L.G., Tannenbaum S.R. (2009). Liver tissue engineering in the evaluation of drug safety. Expert Opin. Drug Metab. Toxicol..

[28] Langer R., Vacanti J.P. (1993). Tissue Engineering. Science.

[29] Nakamura M., Mir T., Arai K., Ito S., Yoshida T., Iwanaga S., Kitano H., Obara C., Nikaido T. (2015). Bioprinting with pre-cultured cellular constructs towards tissue engineering of hierarchical tissues. Int. J. Bioprinting.

[30] Dutta R.C., Dutta A.K. (2010). Comprehension of ECM-cell dynamics: A prerequisite for tissue regeneration. Biotechnol. Adv..

[31] Li Y.-S., Harn H.-J., Hsieh D.-K., Wen T.-C., Subeq Y.-M., Sun L.-Y., Lin S.-Z., Chiou T.-W. (2013). Cells and materials for liver tissue engineering. Cell Transplant..

[32] Arai K., Tsukamoto Y., Yoshida H., Sanae H., Ahmad Mir T., Sakai S., Yoshida T., Okabe M., Nikaido T., Taya M. (2016). The development of cell-adhesive hydrogel for 3D printing. Int. J. Bioprint..

[33] Geckil H., Xu F., Zhang X., Moon S., Demirci U. (2010). Engineering hydrogels as extracellular matrix mimics. Nanomedicine.

[34] Mir T.A., Nakamura M. (2017). Three-dimensional bioprinting: Toward the era of manufacturing human organs as spare parts for healthcare and medicine. Tissue Eng. Part B Rev..

[35] Bahram M., Mohseni N., Moghtader M. (2016). Emerging Concepts in Analysis and An Introduction to Hydrogels and Some Recent Applications of Hydrogels.

[36] Lee K.Y., Mooney D.J. (2001). Hydrogels for Tissue Engineering. Chem. Rev..

[37] Drury J.L., Mooney D.J. (2003). Hydrogels for tissue engineering: Scaffold design variables and applications. Biomaterials.

[38] Kumar A., Han S.S. (2017). PVA-based hydrogels for tissue engineering: A review. Int. J. Polym. Mater. Polym. Biomater..

[39] Dong C., Lv Y. (2016). Application of Collagen Scaffold in Tissue Engineering: Recent Advances and New Perspectives. Polymers.

[40] Cruz-Acuña R., García A.J. (2017). Synthetic hydrogels mimicking basement membrane matrices to promote cell-matrix interactions. Matrix Biol..

[41] Annabi N., Tamayol A., Uquillas J.A., Akbari M., Bertassoni L.E., Cha C., Camci-Unal G., Dokmeci M.R., Peppas N.A., Khademhosseini A. (2014). 25th anniversary article: Rational design and applications of hydrogels in regenerative medicine. Adv. Mater..

[42] Bao J., Shi Y., Sun H., Yin X., Yang R., Li L., Chen X., Bu H. (2011). Construction of a portal implantable functional tissue-engineered liver using perfusion-decellularized matrix and hepatocytes in rats. Cell Transplant..

[43] Baptista P.M., Siddiqui M.M., Lozier G., Rodriguez S.R., Atala A., Soker S. (2011). The use of whole organ decellularization for the generation of a vascularized liver organoid. Hepatology.

[44] Agarwal T., Maiti T.K., Ghosh S.K. (2019). Decellularized caprine liver-derived biomimetic and pro-angiogenic scaffolds for liver tissue engineering. Mater. Sci. Eng. C Mater. Biol. Appl..

[45] Nakamura S., Ijima H. (2013). Solubilized matrix derived from decellularized liver as a growth factor-immobilizable scaffold for hepatocyte culture. J. Biosci. Bioeng..

[46] Shirakigawa N., Ijima H., Takei T. (2012). Decellularized liver as a practical scaffold with a vascular network template for liver tissue engineering. J. Biosci. Bioeng..

[47] Antarianto R.D., Pragiwaksana A., Septiana W.L., Mazfufah N.F., Mahmood A. (2022). Hepatocyte Differentiation from iPSCs or MSCs in Decellularized Liver Scaffold: Cell-ECM Adhesion, Spatial Distribution, and Hepatocyte Maturation Profile. Organogenesis.

[48] Nooshin B.J., Tayebi T., Babajani A., Khani M.M., Niknejad H. (2023). Effects of Different Perfusing Routes through The Portal Vein, Hepatic Vein, and Biliary Duct on Whole Rat Liver Decellularization. Cell J..

[49] Shepherd E.L., Northall E., Papakyriacou P., Safranska K., Sorensen K.K., Lalor P.F. (2023). Decellularization of the Human Liver to Generate Native Extracellular Matrix for Use in Automated Functional Assays with Stellate Cells. Hepatic Stellate Cells: Methods and Protocols.

[50] Rezakhani S., Gjorevski N., Lutolf M.P. (2021). Extracellular matrix requirements for gastrointestinal organoid cultures. Biomaterials.

[51] Martinez-Hernandez A., Amenta P.S. (1993). The hepatic extracellular matrix. Components and distribution in normal liver. Virchows Arch. A Pathol. Anat. Histopathol..

[52] Jin Y., Zhang J., Xu Y., Yi K., Li F., Zhou H., Wang H., Chan H.F., Lao Y.-H., Lv S. (2023). Stem Cell-Derived Hepatocyte Therapy Using Versatile Biomimetic Nanozyme Incorporated Nanofiber-Reinforced Decellularized Extracellular Matrix Hydrogels for the Treatment of Acute Liver Failure. Bioact. Mater..

[53] Martinez-Hernandez A. (1984). The hepatic extracellular matrix. Electron immunohistochemical studies in normal rat liver. Lab. Investig..

[54] Bedossa P., Paradis V. (2003). Liver extracellular matrix in health and disease. J. Pathol..

[55] Zhu C., Coombe D.R., Zheng M.H., Yeoh G.C.T., Li L. (2013). Liver progenitor cell interactions with the extracellular matrix. J. Tissue Eng. Regen. Med..

[56] Maher J.J., Bissell D.M. (1993). Cell-matrix interactions in liver. Semin. Cell Biol..

[57] McQuitty C.E., Williams R., Chokshi S., Urbani L. (2020). Immunomodulatory Role of the Extracellular Matrix Within the Liver Disease Microenvironment. Front. Immunol..

[58] Villesen I.F., Daniels S.J., Leeming D.J., Karsdal M.A., Nielsen M.J. (2020). Review article: The signalling and functional role of the extracellular matrix in the development of liver fibrosis. Aliment. Pharmacol. Ther..

[59] Bissell D.M., Choun M.O. (1988). The role of extracellular matrix in normal liver. Scand. J. Gastroenterol. Suppl..

[60] Bruckner P. (2010). Suprastructures of extracellular matrices: Paradigms of functions controlled by aggregates rather than molecules. Cell Tissue Res..

[61] Mouw J.K., Ou G., Weaver V.M. (2014). Extracellular matrix assembly: A multiscale deconstruction. Nat. Rev. Mol. Cell Biol..

[62] Jhala D., Vasita R. (2014). A Review on Extracellular Matrix Mimicking Strategies for an Artificial Stem Cell Niche. Polym. Rev..

[63] Mir T.A., Nakamura M., Sakai S. (2023). Mammalian-specific decellularized matrices derived bioink for bioengineering of liver tissue analogues: A review. Int. J. Bioprint..

[64] Badylak S.F., Taylor D., Uygun K. (2011). Whole-organ tissue engineering: Decellularization and recellularization of three-dimensional matrix scaffolds. Annu. Rev. Biomed. Eng..

[65] Yamada K.M., Doyle A.D., Lu J. (2022). Cell-3D matrix interactions: Recent advances and opportunities. Trends Cell Biol..

[66] Zhu L., Yuhan J., Yu H., Zhang B., Huang K., Zhu L. (2023). Decellularized Extracellular Matrix for Remodeling Bioengineering Organoid’s Microenvironment. Small.

[67] Yang W., Wang X., Wang Z. (2022). Engineered liver tissue in vitro to mimic liver functions and its biomedical applications. Mater. Adv..

[68] Karamanos N.K., Theocharis A.D., Neill T., Iozzo R.V. (2019). Matrix modeling and remodeling: A biological interplay regulating tissue homeostasis and diseases. Matrix Biol..

[69] Park K.M., Shin Y.M., Kim K., Shin H. (2018). Tissue Engineering and Regenerative Medicine 2017: A Year in Review. Tissue Eng. Part B Rev..

[70] Uygun B.E., Yarmush M.L., Uygun K. (2012). Application of whole-organ tissue engineering in hepatology. Nat. Rev. Gastroenterol. Hepatol..

[71] Navarro-Tableros V., Herrera Sanchez M.B., Figliolini F., Romagnoli R., Tetta C., Camussi G. (2015). Recellularization of rat liver scaffolds by human liver stem cells. Tissue Eng. Part A..

[72] Moran E.C., Dhal A., Vyas D., Lanas A., Soker S., Baptista P.M. (2014). Whole-organ bioengineering: Current tales of modern alchemy. Transl. Res..

[73] Thanapirom K., Frenguelli L., Al-Akkad W., Zhang Z., Pinzani M., Mazza G., Rombouts K. (2021). Optimization and Validation of a Novel Three-Dimensional Co-Culture System in Decellularized Human Liver Scaffold for the Study of Liver Fibrosis and Cancer. Cancers.

[74] Taylor D.A., Sampaio L.C., Ferdous Z., Gobin A.S., Taite L.J. (2018). Decellularized matrices in regenerative medicine. Acta Biomater..

[75] Croce S., Peloso A., Zoro T., Avanzini M.A., Cobianchi L. (2019). A Hepatic Scaffold from Decellularized Liver Tissue: Food for Thought. Biomolecules.

[76] Amin A., Panayotova G., Guarrera J.V. (2022). Hypothermic machine perfusion for liver graft preservation. Curr. Opin. Organ Transplant..

[77] Minami T., Ishii T., Yasuchika K., Fukumitsu K., Ogiso S., Miyauchi Y., Kojima H., Kawai T., Yamaoka R., Oshima Y. (2019). Novel hybrid three-dimensional artificial liver using human induced pluripotent stem cells and a rat decellularized liver scaffold. Regen. Ther..

[78] Acun A., Oganesyan R., Uygun K., Yeh H., Yarmush M.L., Uygun B.E. (2021). Liver donor age affects hepatocyte function through age-dependent changes in decellularized liver matrix. Biomaterials.

[79] Brown M., Li J., Moraes C., Tabrizian M., Li-Jessen N.Y.K. (2022). Decellularized extracellular matrix: New promising and challenging biomaterials for regenerative medicine. Biomaterials.

[80] Zhang X., Chen X., Hong H., Hu R., Liu J., Liu C. (2022). Decellularized extracellular matrix scaffolds: Recent trends and emerging strategies in tissue engineering. Bioact. Mater..

[81] Prestwich G.D. (2008). Evaluating drug efficacy and toxicology in three dimensions: Using synthetic extracellular matrices in drug discovery. Acc. Chem. Res..

[82] Ren H., Shi X., Tao L., Xiao J., Han B., Zhang Y., Yuan X., Ding Y. (2013). Evaluation of two decellularization methods in the development of a whole-organ decellularized rat liver scaffold. Liver Int..

[83] Mirmalek-Sani S.H., Sullivan D.C., Zimmerman C., Shupe T.D., Petersen B.E. (2013). Immunogenicity of decellularized porcine liver for bioengineered hepatic tissue. Am. J. Pathol..

[84] Pan M.X., Hu P.Y., Cheng Y., Cai L.Q., Rao X.H., Wang Y., Gao Y. (2014). An efficient method for decellularization of the rat liver. J. Formos. Med. Assoc..

[85] Ye J.-S., Stoltz J.-F., de Isla N., Liu Y., Yin Y.-F., Zhang L. (2015). An approach to preparing decellularized whole liver organ scaffold in rat. Biomed. Mater. Eng..

[86] Wang Y., Bao J., Wu Q., Zhou Y., Li Y., Wu X., Shi Y., Li L., Bu H. (2015). Method for perfusion decellularization of porcine whole liver and kidney for use as a scaffold for clinical-scale bioengineering engrafts. Xenotransplantation.

[87] Struecker B., Hillebrandt K.H., Voitl R., Butter A., Schmuck R.B., Reutzel-Selke A., Geisel D., Joehrens K., Pickerodt P.A., Raschzok N. (2015). Porcine liver decellularization under oscillating pressure conditions: A technical refinement to improve the homogeneity of the decellularization process. Tissue Eng. Part C Methods.

[88] Bao J., Wu Q., Sun J., Zhou Y., Wang Y., Jiang X., Li L., Shi Y., Bu H. (2015). Hemocompatibility improvement of perfusion-decellularized clinical-scale liver scaffold through heparin immobilization. Sci. Rep..

[89] Ogiso S., Yasuchika K., Fukumitsu K., Ishii T., Kojima H., Miyauchi Y., Yamaoka R., Komori J., Katayama H., Kawai T. (2016). Efficient recellularisation of decellularised whole-liver grafts using biliary tree and foetal hepatocytes. Sci. Rep..

[90] Zhou P., Huang Y., Guo Y., Wang L., Ling C., Guo Q., Wang Y., Zhu S., Fan X., Zhu M. (2016). Decellularization and Recellularization of Rat Livers With Hepatocytes and Endothelial Progenitor Cells. Artif. Organs..

[91] Maghsoudlou P., Georgiades F., Smith H., Milan A., Shangaris P., Urbani L., Loukogeorgakis S.P., Lombardi B., Mazza G., Hagen C. (2016). Optimization of Liver Decellularization Maintains Extracellular Matrix Micro-Architecture and Composition Predisposing to Effective Cell Seeding. PLoS ONE.

[92] Bao J., Wu Q., Wang Y., Li Y., Li L., Chen F., Wu X., Xie M., Bu H. (2016). Enhanced hepatic differentiation of rat bone marrow-derived mesenchymal stem cells in spheroidal aggregate culture on a decellularized liver scaffold. Int. J. Mol. Med..

[93] Mazza G., Al-Akkad W., Telese A., Longato L., Urbani L., Robinson B., Hall A., Kong K., Frenguelli L., Marrone G. (2017). Rapid production of human liver scaffolds for functional tissue engineering by high shear stress oscillation-decellularization. Sci. Rep..

[94] Verstegen M.M.A., Willemse J., van den Hoek S., Kremers G.-J., Luider T.M., van Huizen N.A., Willemssen F.E.J.A., Metselaar H.J., IJzermans J.N.M., van der Laan L.J.W. (2017). Decellularization of Whole Human Liver Grafts Using Controlled Perfusion for Transplantable Organ Bioscaffolds. Stem Cells Dev..

[95] Struecker B., Butter A., Hillebrandt K., Polenz D., Reutzel-Selke A., Tang P., Lippert S., Leder A., Rohn S., Geisel D. (2017). Improved rat liver decellularization by arterial perfusion under oscillating pressure conditions. Tissue Eng. Regen. Med..

[96] Coronado R.E., Somaraki-Cormier M., Natesan S., Christy R.J., Ong J.L., Halff G.A. (2017). Decellularization and Solubilization of Porcine Liver for Use as a Substrate for Porcine Hepatocyte Culture: Method Optimization and Comparison. Cell Transplant..

[97] Mattei G., Magliaro C., Pirone A., Ahluwalia A. (2017). Decellularized Human Liver Is Too Heterogeneous for Designing a Generic Extracellular Matrix Mimic Hepatic Scaffold. Artif. Organs.

[98] Ghiringhelli M., Zenobi A., Brizzola S., Gandolfi F., Bontempo V., Rossi S., Brevini T.A.L., Acocella F. (2018). Simple and Quick Method to Obtain a Decellularized, Functional Liver Bioscaffold. Methods Mol. Biol..

[99] Kojima H., Yasuchika K., Fukumitsu K., Ishii T., Ogiso S., Miyauchi Y., Yamaoka R., Kawai T., Katayama H., Yoshitoshi-Uebayashi E.Y. (2018). Establishment of practical recellularized liver graft for blood perfusion using primary rat hepatocytes and liver sinusoidal endothelial cells. Am. J. Transplant..

[100] Chen Y., Devalliere J., Bulutoglu B., Yarmush M.L., Uygun B.E. (2019). Repopulation of intrahepatic bile ducts in engineered rat liver grafts. Technology.

[101] Ansari T., Southgate A., Obiri-Yeboa I., Jones L.G., Greco K., Olayanju A., Mbundi L., Somasundaram M., Davidson B., Sibbons P.D. (2020). Development and Characterization of a Porcine Liver Scaffold. Stem Cells Dev..

[102] Kim D.-H., Ahn J., Kang H.K., Kim M.-S., Kim N.-G., Kook M.G., Choi S.W., Jeon N.L., Woo H.-M., Kang K.-S. (2021). Development of highly functional bioengineered human liver with perfusable vasculature. Biomaterials.

[103] Li S., Liang C., Jiang W., Deng J., Gu R., Li W., Tian F., Tang L., Sun H. (2021). Tissue-Specific Hydrogels Ameliorate Hepatic Ischemia/Reperfusion Injury in Rats by Regulating Macrophage Polarization via TLR4/NF-κB Signaling. ACS Biomater. Sci. Eng..

[104] Jeong W., Kim M.K., Kang H.W. (2021). Effect of detergent type on the performance of liver decellularized extracellular matrix-based bio-inks. J. Tissue Eng..

[105] Janani G., Mandal B.B. (2021). Mimicking Physiologically Relevant Hepatocyte Zonation Using Immunomodulatory Silk Liver Extracellular Matrix Scaffolds toward a Bioartificial Liver Platform. ACS Appl. Mater. Interfaces.

[106] Kang B., Park Y., Hwang D.G., Kim D., Yong U., Lim K.S., Jang J. (2022). Facile Bioprinting Process for Fabricating Size-Controllable Functional Microtissues Using Light-Activated Decellularized Extracellular Matrix-Based Bioinks. Adv. Mater. Technol..

[107] Tomofuji K., Fukumitsu K., Kondo J., Horie H., Makino K., Wakama S., Ito T., Oshima Y., Ogiso S., Ishii T. (2022). Liver ductal organoids reconstruct intrahepatic biliary trees in decellularized liver grafts. Biomaterials.

[108] Willemse J., van Tienderen G., van Hengel E., Schurink I., van der Ven D., Kan Y., de Ruiter P., Rosmark O., Westergren-Thorsson G G., Schneeberger K. (2022). Hydrogels derived from decellularized liver tissue support the growth and differentiation of cholangiocyte organoids. Biomaterials.

[109] Chen J., Ma S., Yang H., Liang X., Yao H., Guo B., Chen D., Jiang J., Shi D., Xin J. (2023). Generation and metabolomic characterization of functional ductal organoids with biliary tree networks in decellularized liver scaffolds. Bioact. Mater..

[110] Van Tienderen G.S., Conboy J., Muntz I., Willemse J., Tieleman J., Monfils K., Schurink I.J., Demmers J.A.A., Doukas M., Koenderink G.H. (2023). Tumor decellularization reveals proteomic and mechanical characteristics of the extracellular matrix of primary liver cancer. Biomater. Adv..

[111] Agarwal T., Narayan R., Maji S., Ghosh S.K., Maiti T.K. (2018). Decellularized caprine liver extracellular matrix as a 2D substrate coating and 3D hydrogel platform for vascularized liver tissue engineering. J. Tissue Eng. Regen. Med..

[112] Saheli M., Sepantafar M., Pournasr B., Farzaneh Z., Vosough M., Piryaei A., Baharvand H. (2018). Three-dimensional liver-derived extracellular matrix hydrogel promotes liver organoids function. J. Cell. Biochem..

[113] Barakat O., Abbasi S., Rodriguez G., Rios J., Wood R.P., Ozaki C., Holley L.S., Gauthier P.K. (2012). Use of decellularized porcine liver for engineering humanized liver organ. J. Surg. Res..

[114] Tajima K., Yagi H., Kitagawa Y. (2018). Human-Scale Liver Harvest and Decellularization for Preclinical Research. Methods Mol. Biol..

[115] Scarritt M.E., Pashos N.C., Bunnell B.A. (2015). A review of cellularization strategies for tissue engineering of whole organs. Front. Bioeng. Biotechnol..

[116] Fu R.-H., Wang Y.-C., Liu S.-P., Shih T.-R., Lin H.-L., Chen Y.-M., Sung J.-H., Lu C.-H., Wei J.-R., Wang Z.-W. (2014). Decellularization and recellularization technologies in tissue engineering. Cell Transplant..

[117] Soto-Gutierrez A., Zhang L., Medberry C., Fukumitsu K., Jiang H., Gramignoli R., Komori J., Ross M., Nagaya M., Lagasse E. (2011). A whole-organ regenerative medicine approach for liver replacement. Tissue Eng. Part C Methods..

[118] Afzal Z., Huguet E.L. (2023). Bioengineering liver tissue by repopulation of decellularised scaffolds. World J. Hepatol..

[119] Lee E., Kim H.J., Shaker M.R., Ryu J.R., Ham M.S., Seo S.H., Kim D.H., Lee K., Jung N., Choe Y. (2019). High-Performance Acellular Tissue Scaffold Combined with Hydrogel Polymers for Regenerative Medicine. ACS Biomater. Sci. Eng..

[120] Hughes O.B., Rakosi A., Macquhae F., Herskovitz I., Fox J.D., Kirsner R.S. (2016). A review of cellular and acellular matrix products: Indications, techniques, and outcomes. Plast. Reconstr. Surg..

[121] Wang J., Qin X., Xia S., Liu S., Ren H. (2023). Orthotopic implantable liver decellularized scaffold for acute liver failure. Eng. Regen..

[122] White L.J., Taylor A.J., Faulk D.M. (2017). The impact of detergents on the tissue decellularization process: A ToF-SIMS study. Acta Biomater..

[123] Zambon J.P., Atala A., Yoo J.J. (2020). Methods to generate tissue-derived constructs for regenerative medicine applications. Methods.

[124] Choudhury D., Yee M., Sheng Z.L.J., Amirul A., Naing M.W. (2020). Decellularization systems and devices: State-of-the-art. Acta Biomater..

[125] Walraven M., Hinz B. (2018). Therapeutic approaches to control tissue repair and fibrosis: Extracellular matrix as a game changer. Matrix Biol..

[126] Wang B., Li W., Dean D., Mishra M.K., Wekesa K.S. (2018). Enhanced hepatogenic differentiation of bone marrow derived mesenchymal stem cells on liver ECM hydrogel. J. Biomed. Mater. Res. A.

[127] Park K.-M., Hussein K.H., Hong S.-H., Ahn C., Yang S.-R., Park S.-M., Kweon O.-K., Kim B.-M., Woo H.-M. (2016). Decellularized Liver Extracellular Matrix as Promising Tools for Transplantable Bioengineered Liver Promotes Hepatic Lineage Commitments of Induced Pluripotent Stem Cells. Tissue Eng. Part A.

[128] Loneker A.E., Faulk D.M., Hussey G.S., D’Amore A., Badylak S.F. (2016). Solubilized liver extracellular matrix maintains primary rat hepatocyte phenotype in-vitro. J. Biomed. Mater. Res. A.

[129] Kajbafzadeh A.M., Farazmand N.J., Monajemzadeh M., Baghayee A. (2013). Determining the optimal decellularization and sterilization protocol for preparing a tissue scaffold of a human-sized liver tissue. Tissue Eng. Part C Methods.

[130] Ott H.C., Matthiesen T.S., Goh S.K., Black L.D., Kren S.M., Netoff T.I., Taylor D.A. (2008). Perfusion-decellularized matrix: Using nature’s platform to engineer a bioartificial heart. Nat. Med..

[131] Wang B., Qinglai T., Yang Q., Li M., Zeng S., Yang X., Xiao Z., Tong X., Lei L., Li S. (2023). Functional acellular matrix for tissue repair. Mater. Today Biol..

[132] Lin P., Chan W.C.N., Badylak S.F., Bhatia S.N. (2004). Assessing porcine liver-derived biomatrix for hepatic tissue engineering. Tissue Eng..

[133] Lewis P.L., Yan M., Su J., Shah R.N. (2019). Directing the growth and alignment of biliary epithelium within extracellular matrix hydrogels. Acta Biomater..

[134] Roth S.P., Erbe I., Burk J., Turksen K. (2018). Decellularized Scaffolds and Organogenesis: Methods and Protocols.

[135] Toprakhisar B., Verfaillie C.M., Kumar M. (2023). Advances in Recellularization of Decellularized Liver Grafts with Different Liver (Stem) Cells: Towards Clinical Applications. Cells.

[136] Gao Y., Li Z., Hong Y., Li T., Hu X., Sun L., Chen Z., Chen Z., Luo Z., Wang X. (2020). Decellularized liver as a translucent ex vivo model for vascular embolization evaluation. Biomaterials.

[137] Dias M.L., Paranhos B.A., Goldenberg R.C.S. (2022). Liver scaffolds obtained by decellularization: A transplant perspective in liver bioengineering. J. Tissue Eng..

[138] Meng F., Assiri A., Dhar D., Broering D.C. (2017). Whole liver engineering: A promising approach to develop functional liver surrogates. Liver Int..

[139] Naeem E.M., Sajad D., Talaei-Khozani T., Khajeh S., Azarpira N., Alaei S., Tanideh N., Reza T.M., Razban V. (2019). Decellularized liver transplant could be recellularized in rat partial hepatectomy model. J. Biomed. Mater. Res. A.

[140] Hussein K.H., Park K.M., Yu L., Song S.-H., Woo H.-M., Kwak H.-H. (2020). Vascular reconstruction: A major challenge in developing a functional whole solid organ graft from decellularized organs. Acta Biomater..

[141] Rossi E.A., Quintanilha L.F., Nonaka C.K.V., Souza B.S.d.F. (2019). Advances in Hepatic Tissue Bioengineering with Decellularized Liver Bioscaffold. Stem Cells Int..

[142] Uygun B.E., Soto-Gutierrez A., Yagi H., Izamis M.-L., Guzzardi M.A., Shulman C., Milwid J., Kobayashi N., Tilles A., Berthiaume F. (2010). Organ reengineering through development of a transplantable recellularized liver graft using decellularized liver matrix. Nat. Med..

[143] Shupe T., Williams M., Brown A., Willenberg B., Petersen B.E. (2010). Method for the decellularization of intact rat liver. Organogenesis.

[144] Gessner R.C., Hanson A.D., Feingold S., Cashion A.T., Corcimaru A., Wu B.T., Mullins C.R., Aylward S.R., Reid L.M., Dayton P.A. (2013). Functional ultrasound imaging for assessment of extracellular matrix scaffolds used for liver organoid formation. Biomaterials.

[145] Yagi H., Fukumitsu K., Fukuda K., Kitago M., Shinoda M., Obara H., Itano O., Kawachi S., Tanabe M., Coudriet G.M. (2013). Human-scale whole-organ bioengineering for liver transplantation: A regenerative medicine approach. Cell Transplant..

[146] Ko I.K., Peng L., Peloso A., Smith C.J., Dhal A., Deegan D.B., Zimmerman C., Clouse C., Zhao W., Shupe T.D. (2015). Bioengineered transplantable porcine livers with re-endothelialized vasculature. Biomaterials.

[147] Hussein K.H., Park K.M., Kang K.S., Woo H.M. (2016). Heparin-gelatin mixture improves vascular reconstruction efficiency and hepatic function in bioengineered livers. Acta Biomater..

[148] Devalliere J., Chen Y., Dooley K., Yarmush M.L., Uygun B.E. (2018). Improving functional re-endothelialization of acellular liver scaffold using REDV cell-binding domain. Acta Biomater..

[149] Meng F., Almohanna F., Altuhami A., Assiri A.M., Broering D.C. (2019). Vasculature reconstruction of decellularized liver scaffolds via gelatin-based re-endothelialization. J. Biomed. Mater. Res. A.

[150] Takeishi K., Collin de l’Hortet A., Wang Y., Handa K., Guzman-Lepe J., Matsubara K., Morita K., Jang S., Haep N., Florentino R.M. (2020). Assembly and Function of a Bioengineered Human Liver for Transplantation Generated Solely from Induced Pluripotent Stem Cells. Cell Rep..

[151] Chen C., Gutierrez A.S., Baptista P.M., Spee B. (2018). Biotechnology Challenges to In Vitro Maturation of Hepatic Stem Cells. Gastroenterology.

[152] Baxter M., Withey S., Harrison S., Segeritz C.-P., Zhang F., Atkinson-Dell R., Rowena S.-Y., Gerrard D.T., Sison-Young R., Jenkins R. (2015). Phenotypic and functional analyses show stem cell-derived hepatocyte-like cells better mimic fetal rather than adult hepatocytes. J. Hepatol..

[153] Ogawa M., Ogawa S., Bear C.E., Ahmadi S., Chin S., Li B., Grompe M., Keller G., Kamath B.M., Ghanekar A. (2015). Directed differentiation of cholangiocytes from human pluripotent stem cells. Nat. Biotechnol..

[154] Shaheen M.F., Joo D.J., Ross J.J., Anderson B.D., Chen H.S., Huebert R.C., Li Y., Amiot B., Young A., Zlochiver V. (2020). Sustained perfusion of revascularized bioengineered livers heterotopically transplanted into immunosuppressed pigs. Nat. Biomed. Eng..

[155] Anderson B.D., Nelson E.D., Joo D., Amiot B.P., Katane A.A., Mendenhall A., Steiner B.G., Stumbras A.R., Nelson V.L., Palumbo R.N. (2021). Functional characterization of a bioengineered liver after heterotopic implantation in pigs. Commun. Biol..

[156] Higashi H., Yagi H., Kuroda K., Tajima K., Kojima H., Nishi K., Morisaku T., Hirukawa K., Fukuda K., Matsubara K. (2022). Transplantation of bioengineered liver capable of extended function in a preclinical liver failure model. Am. J. Transplant..

[157] Khajavi M., Hashemi M., Kalalinia F. (2021). Recent advances in optimization of liver decellularization procedures used for liver regeneration. Life Sci..

[158] Dai Q., Jiang W., Huang F., Song F., Zhang J., Zhao H. (2022). Recent Advances in Liver Engineering With Decellularized Scaffold. Front. Bioeng. Biotechnol..

[159] Hosseini V., Maroufi N.F., Saghati S., Asadi N., Darabi M., Ahmad S.N.S., Hosseinkhani H., Rahbarghazi R. (2019). Current progress in hepatic tissue regeneration by tissue engineering. J. Transl. Med..

